# Precise positioning with current multi-constellation Global Navigation Satellite Systems: GPS, GLONASS, Galileo and BeiDou

**DOI:** 10.1038/srep08328

**Published:** 2015-02-09

**Authors:** Xingxing Li, Xiaohong Zhang, Xiaodong Ren, Mathias Fritsche, Jens Wickert, Harald Schuh

**Affiliations:** 1 School of Geodesy and Geomatics, Wuhan University, 129 Luoyu Road, 430079, Wuhan, Hubei, China; 2 German Research Centre for Geosciences (GFZ), Telegrafenberg, 14473 Potsdam, Germany

## Abstract

The world of satellite navigation is undergoing dramatic changes with the rapid development of multi-constellation Global Navigation Satellite Systems (GNSSs). At the moment more than 70 satellites are already in view, and about 120 satellites will be available once all four systems (BeiDou + Galileo + GLONASS + GPS) are fully deployed in the next few years. This will bring great opportunities and challenges for both scientific and engineering applications. In this paper we develop a four-system positioning model to make full use of all available observations from different GNSSs. The significant improvement of satellite visibility, spatial geometry, dilution of precision, convergence, accuracy, continuity and reliability that a combining utilization of multi-GNSS brings to precise positioning are carefully analyzed and evaluated, especially in constrained environments.

Over the past decades, the Global Positioning System (GPS), as the first space-based radio-navigation system comprised of a dedicated satellite constellation, has made remarkable contributions to scientific applications (e.g., geodesy, remote sensing, space and fundamental physics) and engineering services (e.g., surveying, navigation, and timing)^1^,^2^,^3^. Currently, with two new and emerging constellations (BeiDou, Galileo) as well as the recovery of Russia's GLONASS, the world of satellite navigation is undergoing dramatic changes with excellent potential for extended and more precise and reliable GNSS applications and services^4^.

The GLONASS constellation has been fully recovered since October 2011 and is operating at full capability with 24 satellites in orbits at the moment, enabling full global coverage (http://www.glonass-ianc.rsa.ru/en/GLONASS/). Europe's Galileo is the third GNSS, aiming to offer a continuous, more flexible and precise positioning service with a whole set of related parameters and sub-services to all ranges of users. At the moment it is in its In-Orbit Validation (IOV) phase. As part of this phase four IOV satellites have been launched and are in orbit. Two FOC (full operational capability) satellites were launched on August 22th, 2014, but with wrong orbit parameters. The full Galileo constellation will consist of 30 satellites in three orbital planes, including three in-orbit spare ones (http://www.esa.int/Our_Activities/Navigation/The_future_-_Galileo/What_is_Galileo). The BeiDou navigation satellite system, being established independently in China, is pacing steadily forward towards its final destination - an operational global navigation satellite system comprises 5 GEO (Geostationary Earth Orbit), 3 IGSO (Inclined Geo-Synchronous Orbit), and 27 MEO (Medium Earth Orbit) satellites by 2020. The two-phase schedule enables its rapid emerging with operational services over the Asia-Pacific region now. The first phase consists of five satellites in GEO at an altitude of 35,786 km, five in IGSO at an altitude of 35,786 km as well as with 55° inclination to the equatorial plane, and four in MEO at an altitude of 21,528 km and 55° inclination to the equatorial plane. This phase has been completed by the end of 2012. Hence, the regional positioning and navigation services could be operational provided for users throughout the Asia-Pacific area^5^,^6^. Up to now, 74 satellites are already in view and transmitting data compared to past years with 32 GPS only. Once all four systems are fully deployed, about 120 navigation satellites will be available for GNSS users. Undoubtedly, the rapid development of multi-constellation GNSS could enable a wider range of more precise applications, e.g. for positioning, navigation, timing, and remote sensing^7^.

The International GNSS Service (IGS), as the gold standard for high-precision GPS data analysis, is fully committed to expand to a true multi-GNSS service. It has initiated the Multi-GNSS Experiment (MGEX) to collect and analyze data of GPS, GLONASS, BeiDou and Galileo and also serves as a framework for increasing the overall awareness of multi-GNSS within the scientific and engineering communities^4^. As a backbone of the MGEX project, over the past one to two years a new network of multi-GNSS monitoring stations has been deployed around the globe in parallel to the legacy IGS network for GPS and GLONASS. The MGEX network has grown to more than 90 stations now and it provides an excellent opportunity to demonstrate the potential benefits from multi-constellation GNSS (http://igs.org/mgex/).

The fusion of multiple GNSSs will significantly increase the number of observed satellites, optimize the spatial geometry and improve continuity and reliability of positioning. However, in the past the data fusion of multi-GNSS only focused on GPS and GLONASS^8^,^9^,^10^. Thanks to the completion of the BeiDou regional system, GPS + BeiDou combined precise orbit determination^7^,^11^,^12^,^13^ (POD) and precise point positioning^14^,^15^ (PPP) have been investigated recently. Some initial results about GPS + Galileo combined POD^16^,^17^ and real time kinematic^18^ (RTK) positioning were also presented.

In this contribution, we develop a BeiDou + Galileo + GLONASS + GPS four-system model to fully exploit all the observations from four navigation satellite systems and demonstrate its significant contribution to precise positioning with current multi-constellations. The data processing model and strategy for multi-GNSS precise positioning are described in detail in the ‘Methods’ section. We perform a rigorous multi-GNSS analysis with careful consideration of inter-system and inter-frequency biases. All the observations from different GNSS are processed together in one common parameter estimation procedure with appropriate ionospheric constraints. In the ‘Results’ section, the satellite visibility, positional dilution of precision (PDOP), signal-to-noise ratio (SNR), multipath, and phase and code residuals of different constellations are carefully analyzed and compared to assess their individual performance. Furthermore, a comprehensive analysis, including satellite visibility, spatial geometry, dilution of precision, convergence, accuracy, continuity and reliability, is performed to evaluate the contribution of multi-GNSS fusion to precise positioning, especially in constrained environments (e.g., urban canyons, open pits).

## Results

**Figure 1:**
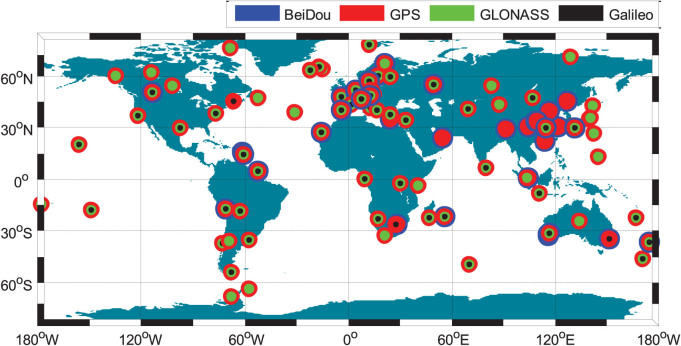
The distribution of multi-GNSS stations from MGEX and BETN networks. Their supported constellations are shown in different colors, BeiDou in blue, GPS in red, GLONASS in green, and Galileo in black. This figure is drawn using GMT software.

In order to assess the precise positioning performance with current multi-constellation GNSS, we analyzed 100 days' data of MGEX and BETN^19^ (BeiDou Experimental Tracking Network) networks from September 1 to December 9 (day of year 244 to 343) in 2013. Figure 1 shows the distribution of MGEX and BETN networks, which includes stations all over the world.

**Figure 2:**
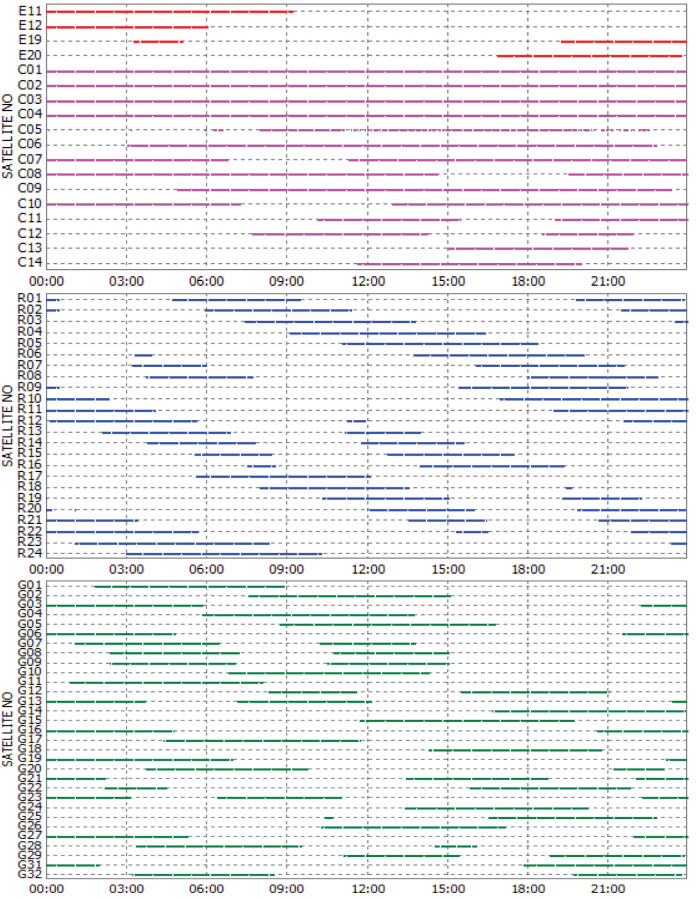
Satellite visibility of Galileo, BeiDou, GLONASS and GPS at the four-system station GMSD (Japan) on September 3, 2013 (GPS Time). Galileo, BeiDou, GLONASS and GPS satellites are shown by red, pink, blue and green lines, respectively.

**Figure 3:**
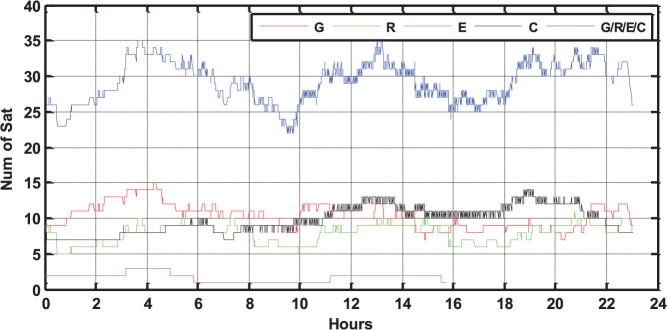
Visible satellite number at the four-system station GMSD on September 3, 2013 (GPS Time). The satellite number of GPS, GLONASS, BeiDou, Galileo and the total satellite number are shown by red, green, pink, black and blue lines, respectively.

Taking the four-system station GMSD (Japan, 30.55°N, 131.01°E) as an example, the satellite visibilities for Galileo, BeiDou, GLONASS and GPS on September 3, 2013 are shown in Figure 2. It is obvious that a joint utilization of various GNSSs brings a significant improvement of satellite visibility. It also can be seen that the visibility is very different for the various orbital types. The BeiDou GEO satellites have the longest tracking periods, e.g., C01 ~ C04 are tracked for the whole day. The BeiDou IGSO satellites C06–C10 are tracked relatively shorter than GEOs with data gaps of about 4.5 h for one day. The tracking periods of MEO satellites are the shortest and about 6 ~ 10 h for one day. The special constellation of BeiDou, including five GEOs and five IGSOs, guarantees sufficient visible satellites in the Asia-Pacific area. The number of visible satellites for each GNSS and the total satellite number of four systems at GMSD on that day are shown in Figure 3. At each epoch, there are about 7 ~ 13 BeiDou, 5 ~ 10 GLONASS, and 7 ~ 14 GPS satellites, which ensures their autonomous positioning. The Galileo satellites in view vary between 0 ~ 3 and therefore this GNSS currently cannot perform autonomous positioning. The blue line shows, that the total satellite number of all four systems can even reach up to about 23 ~ 35 at each epoch. Such a redundancy of the satellite visibility significantly increases the reliability and robustness of precise positioning.

**Figure 4:**
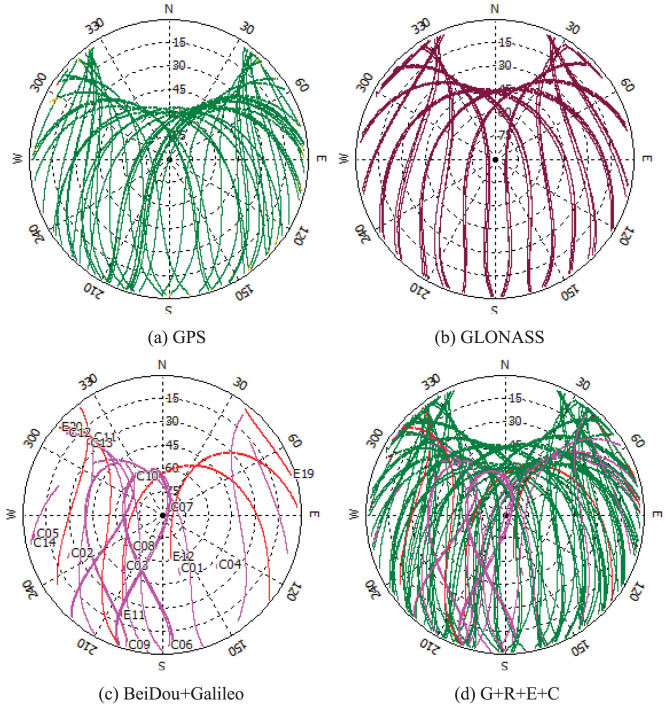
Sky plots (azimuth vs. elevation) for the various satellite systems at GMSD on September 3, 2013. a) GPS; b) GLONASS; c) BeiDou (pink) and Galileo (red); d) all the satellites including GPS, GLONASS, BeiDou and Galileo.

**Figure 5:**
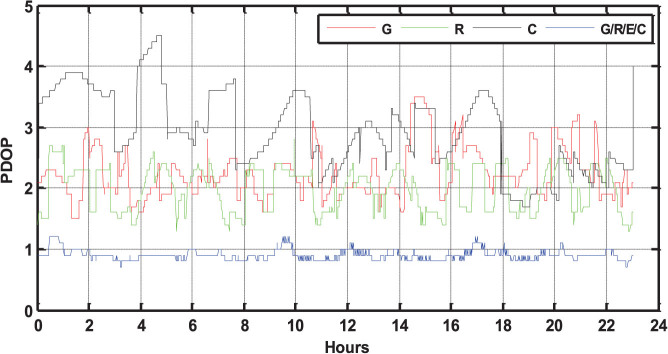
PDOP values for the single-system and four-system modes at GMSD on September 3, 2013 (GPS Time). The PDOP values of GPS, GLONASS, BeiDou and four-system combination are shown by red, green, black, and blue lines, respectively.

Figure 4a, b and c show the sky plots (azimuth vs. elevation) of GPS, GLONASS, Galileo + BeiDou at GMSD, respectively. Compared with Figure 4d, the improvement of the spatial geometry is clearly visible when observations from the four navigation systems are used simultaneously. The PDOP values in single-system and four-system modes on that day are also calculated and shown in Figure 5. The GPS PDOP values vary between 1.5 and 3.5, while the GLONASS PDOP values are about 1.2 ~ 3. The BeiDou PDOP values are larger than GPS and GLONASS, about 1.8 ~ 4.5. The blue line of Figure 5 shows that the PDOP values in four-system mode are significantly reduced to below 1.0 in most of the time and the corresponding time series is very stable.

**Figure 6:**
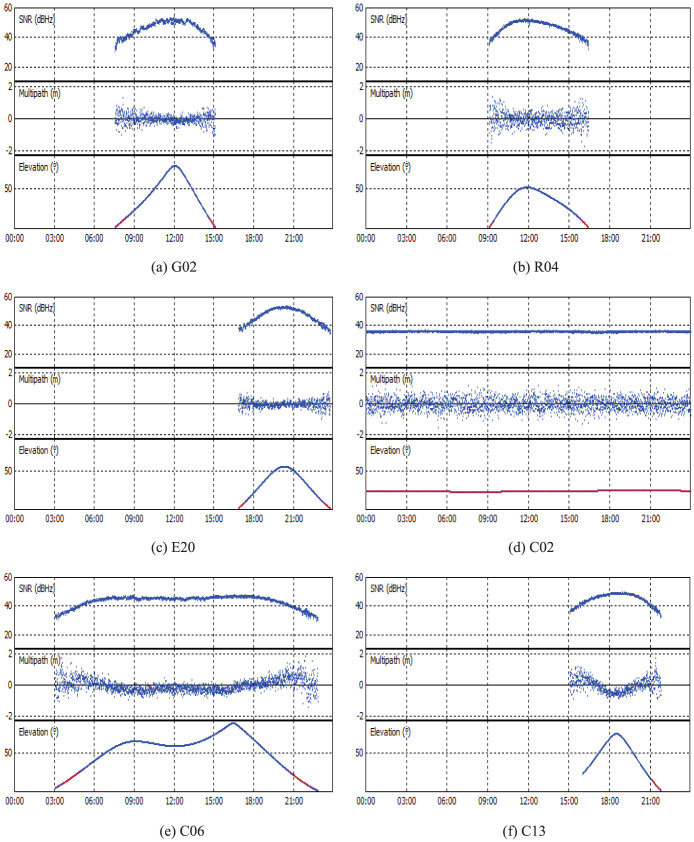
The SNRs and MPCs of different satellite systems and orbital types at GMSD. G02, R04, E20, C02(GEO), C06(IGSO) and C13(MEO) are selected as typical examples for their individual satellite system or orbital type. The variation of their elevations with time (GPS Time) is also shown.

Figure 6 shows the signal-to-noise ratio (SNR) and multipath combination (MPC^12^, which mainly contains code noise and multipath) values at GMSD to assess and compare the observation quality for the different satellite systems. As typical examples, the SNRs and MPCs of the satellites G02, R04 and E20 on the first frequency along with their variation of the elevations are shown in Figure 6a, b and c, respectively. The SNRs and MPCs of BeiDou GEO C02, IGSO C06 and MEO C13 are shown in Figure 6d, e and f, respectively. It can be clearly seen that both the SNR and MPC values are strongly correlated with the elevation variations. The higher the elevation angles are, the larger the SNR and the smaller the MPC values. Generally speaking, the SNR are higher than 40 and the MPC values are smaller than 0.5 m when the elevation angles are higher than 15°. We also find that the different orbital types show different variation characteristics for SNR and MPC. The IGSO satellites can be observed much longer and the corresponding SNR, MPC and elevation values change much slower than for the MEO satellites. Especially, the elevation angles of each GEO satellite stay around one specific value, the corresponding SNR and MPC values are the steadiest and nearly don't exhibit systematic variations.

**Figure 7:**
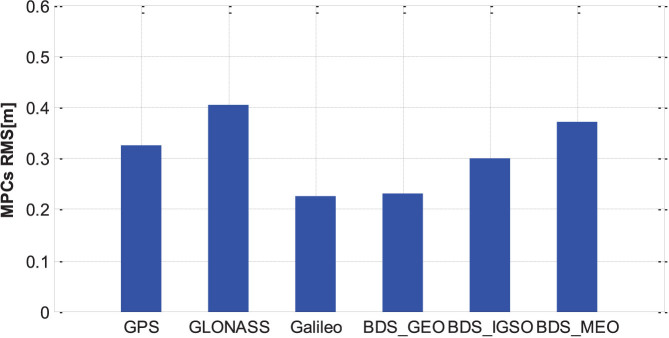
Standard deviations of MPCs for different satellite systems and orbital types at GMSD station. (GPS, GLONASS, Galileo, BeiDou GEO, BeiDou IGSO and BeiDou MEO; on the first frequency).

Figure 7 shows the standard deviations (STD) of the entire MPC series for different satellite systems and orbital types. The STD of the GPS MPC values is about 0.32 m and the STD of the GLONASS MPCs is slightly larger than GPS with about 0.4 m. The STD of the BeiDou MEO's MPC values is about 0.38 m, larger than for GPS and slightly smaller than for GLONASS. Galileo has the smallest STD of the MPC values with about 0.22 m. For the different orbital types of the BeiDou satellites, the GEO MPC values are smallest with a STD about 0.22 m, compared to IGSO with about 0.3 m, which is between GEO and MEO. It can be explained that the proportion of low-elevation periods for IGSO is lower than that of MEO as shown in Figure 6e, and the GEO satellites have the lowest low-elevation proportion as their elevations are always around some relatively high values within their service regions as shown in Figure 6d.

**Figure 8:**
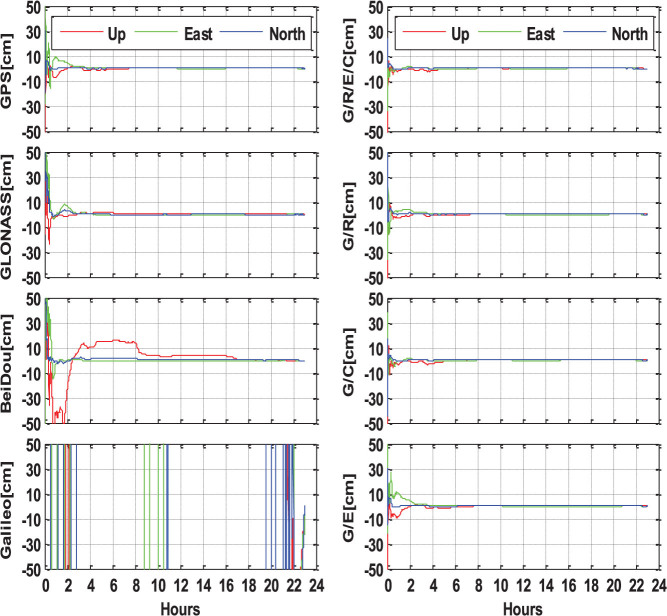
Static PPP solutions of single-system (G, R, E, and C), dual-system (G/R, G/C, and G/E) and four-system (G/R/E/C) modes at station CUT0 (Australia, 32.00°S, 115.89°E, ), on September 3, 2013 (GPS Time). The north, east and up components are shown by the blue, green and red lines, respectively.

The multi-GNSS data from MGEX and BETN networks are processed in both single- and multi-system modes following the positioning algorithms presented in the Section ‘Methods’. All the estimated station coordinates are compared with the IGS SINEX or weekly solution. Figure 8 shows static PPP solutions of single-, dual- and four-system modes at the four-system station CUT0 in Australia, which is covered by the BeiDou service of the Asia-Pacific area. The left sub-figures show the single-system PPP results of GPS-only, BeiDou-only, GLONASS-only and Galileo-only, respectively. For the GPS-only solution, the positioning accuracy can be better than 1 dm after a convergence time of about 30 minutes. About 2 hours of convergence is required to ensure an accuracy of better than 5 cm in all three components. The mm accuracy can be achieved after the long convergence time of several hours. The convergence of GLONASS-only PPP is longer compared to GPS-only PPP, about 3 hours to achieve an accuracy of a few centimeters. Meanwhile, the GLONASS positioning accuracy after sufficient convergence time is also slightly worse compared to the GPS solution. The BeiDou-only PPP presents good performance in the horizontal components, few cm accuracy can be achieved within one hour. However, the vertical component is much more unstable than GPS and GLONASS. A Galileo-only PPP solution cannot be derived at this station as not enough satellites can be observed.

The combined GPS/BeiDou,GPS/GLONASS,GPS/Galileo,GPS + BeiDou + GLONASS + Galileo PPP solutions are shown in the right sub-figures. Obviously, the multi-GNSS combination significantly improves the PPP performance, compared to the left sub-figures of the single-system solutions. It can be clearly seen that the combined GPS/BeiDou and GPS/GLONASS solutions significantly shorten the convergence time and improve the position series compared to single-system PPP. The Galileo satellites also contribute to the combined GPS/Galileo PPP solution to some extent, although they are not sufficient for autonomous positioning. Especially, the combined GPS + BeiDou + GLONASS + Galileo PPP present the fastest convergence, the most stable position series and highest accuracy for all three components. It only takes several minutes to achieve an accuracy of better than 10 cm, less than 30 minutes to be better than 5 cm, and a few hours to reach mm level accuracy.

**Figure 9:**
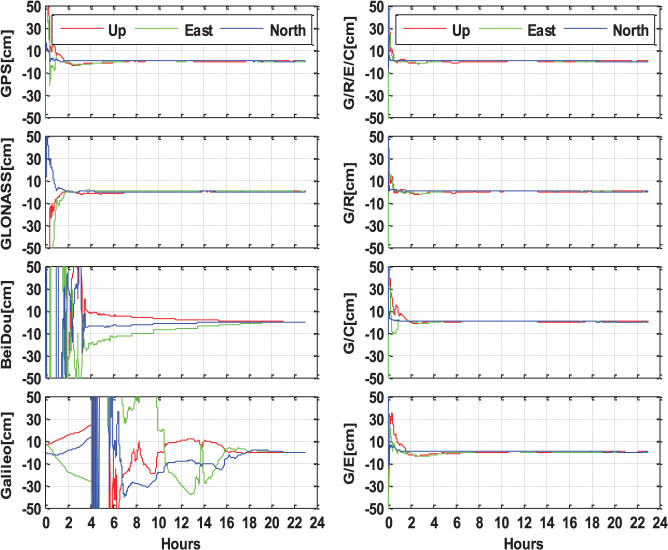
Static PPP solutions of single-system (G, R, E, and C), dual-system (G/R, G/C, and G/E) and four-system (G/R/E/C) modes at station LMMF (14.59°N, −60.99°W, Martinique, Caribbean Sea), on September 3, 2013 (GPS Time). The north, east and up components are shown by the blue, green and red lines, respectively.

Figure 9 shows static PPP solutions at another four-system station LMMF (14.59°N, −60.99°W, Martinique, Caribbean Sea), which is out of the Asia-Pacific region of BeiDou service. At this location, both BeiDou and Galileo cannot provide continuous precise positioning as a stand-alone system. The cm level accuracy for BeiDou-only or Galileo-only PPP is only available after a static observation of even longer than 16 hours. Importantly though, the addition of BeiDou or Galileo to the GPS-only processing, obviously shorten the convergence time and improve the position series. The combined GPS + BeiDou + GLONASS + Galileo PPP also shows the fastest convergence, the most stable position series and highest accuracy in all of the three components.

**Figure 10:**
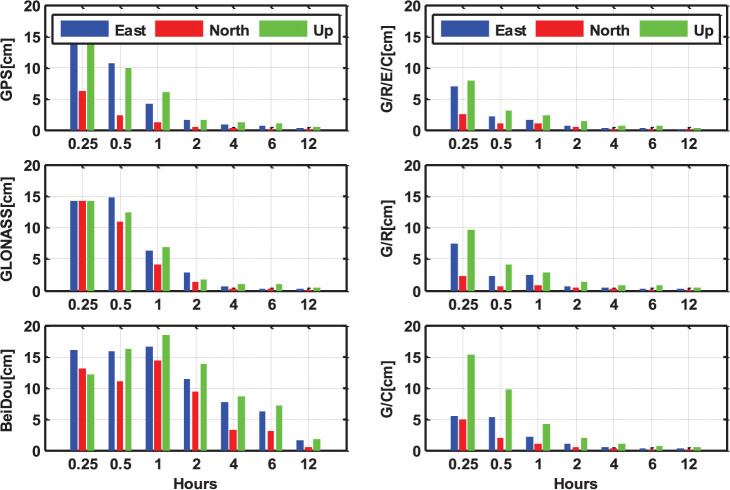
RMS values of static PPP solutions with different session lengths (15 min, 30 min, 1 h, 2 h, 4 h, 6 h and 12 h) in single-, dual- and four-system modes. The north, east and up components are shown by the red, blue and green bars, respectively.

Figure 10 shows the statistical results of the static PPP solutions with different session lengths, of 15 and 30 min, 1, 2, 4, 6, and 12 h. The root mean square (RMS) values are calculated from all static PPP solutions over all the selected days and stations (MGEX and BETN networks from September 1 to December 9 in 2013). The single-system PPP results are shown in the left sub-figures and it can be seen that the positioning accuracy is evidently improved along with the increase of the observational length. With the same session length, the GPS-only PPP can achieve the highest accuracy, while the BeiDou-only PPP has the worst performance. In addition, the positioning accuracy in north component is generally better than east and vertical components. The multi-system PPP solutions are shown in right sub-figures. Obviously, the multi-GNSS combination significantly improves the PPP performance, compared to the left sub-figures of single-system solutions. Usually, single-system PPP requires two hours or longer observation to achieve a positioning accuracy of few centimeters. For multi-GNSS PPP, the accuracy of few centimeters is available within 30 minutes for all three components. With the same session length, multi-PPP accuracy is significantly better than single-system PPP. The north component is still the most accurate component in multi-PPP solutions (may be caused by the satellite constellation configuration). The RMS values of static PPP solutions with different session lengths in single- and four-system modes are listed in Table 1.

**Figure 11:**
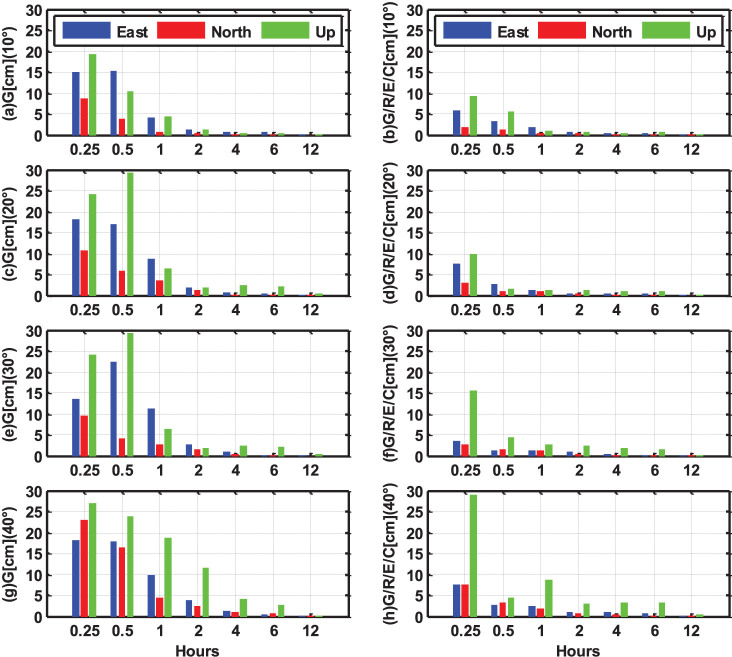
The RMS values of static PPP solutions in GPS-only and four-system modes under different cut-off elevation angles (from 10° to 40°). The north, east and up components are shown by the red, blue and green bars, respectively.

Figure 11 shows the statistical RMS values of static PPP solutions under different cut-off elevation angles, ranging from 10° to 40°. The GPS-only PPP results are shown in the left sub-figures, while the four-system PPP solutions are shown in the right sub-figures. It can be clearly seen that the accuracy of GPS-only PPP decreases significantly when the cut-off elevation angle increases, especially for short session lengths. At the 40° cut-off elevation, the positioning accuracy with 30 min observations is reduced to about 18, 17, and 24 cm in east, north and vertical components, respectively. Even with 2 hours observations, the vertical accuracy is still worse than 10 cm. When the session length is 30 minutes or longer, it can be seen from the right sub-figures that the accuracy of multi-PPP is not obviously affected by high cut-off elevation and few centimeters are achievable in all the three components even with 40° cut-off elevation. Only the vertical accuracy is slightly reduced with the increase of the cut-off elevation angle. With only 15 min observations, the vertical accuracy decreases gradually as the cut-off elevation angle increases, but the horizontal accuracy is only slightly decreased even with 40° cut-off elevation. When the cut-off elevation is increased to 30° or 40°, a static observation of about four hours is required to obtain a positioning accuracy of few centimeters for GPS-only PPP. However, multi-system PPP only needs 30 min static observations to achieve few centimeters accuracy. This is important, since such high cut-off elevation capability will significantly increase the GNSS applicability in constrained environments, such as, e.g., in urban canyons, open pits or when serious low-elevation multipath or ionospheric scintillations are present.

**Figure 12:**
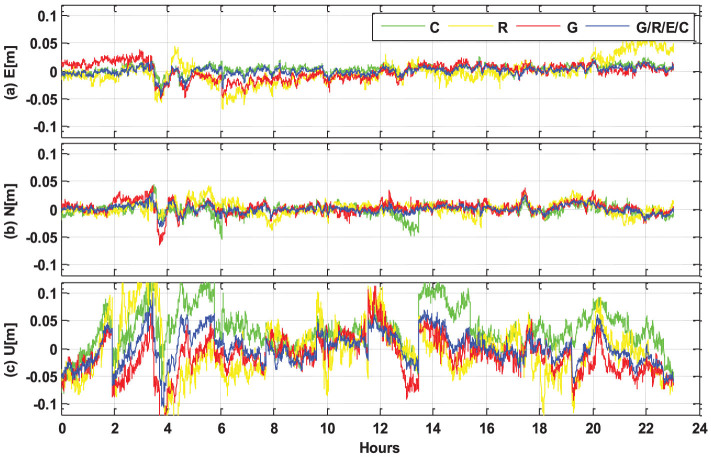
Kinematic PPP results (with backward smoothing) in single- and multi-system modes at station CUT0 (32.00°S, 115.89°E, Australia), on September 3, 2013 (GPS Time). The GPS, GLONASS, BeiDou and four-system solutions are shown by the blue, yellow, green and red lines, respectively.

All the Multi-GNSS data are also processed in kinematic mode, i.e. the station coordinates are estimated epoch-by-epoch without any constraints between the epochs. The kinematic PPP results of single- and four-system modes at station CUT0 are compared in Figure 12 as a typical example. The GPS-only, GLONASS-only and BeiDou-only position series are shown by the blue, yellow and green lines, respectively. The red line shows the four-system PPP results. It is clearly indicated that the position series of multi-PPP is significantly more stable compared to the single-system solutions with much smaller and fewer fluctuations. Several spikes, appearing in single-system solutions, can be removed when multi-GNSS observations are used together.

We calculated the RMS values of kinematic PPP solutions in single- and four-system modes for all the selected days and stations. The statistical results in east, north, and up components are shown in Table 2. For the single-system solutions, GPS has the best accuracy of 1.4, 1.2, and 4.4 cm in the east, north and up components, respectively. The horizontal accuracy of BeiDou-only kinematic PPP is comparable with that of GPS, but the BeiDou vertical accuracy is worse than GPS. The GLONASS-only PPP has the worst performance in all the three components with RMS values of 2.9, 1.8, and 5.9 cm. Compared to the multi-PPP results, one can see that the fusion of multiple GNSS can significantly improve the accuracy of kinematic PPP and the corresponding RMS values are about 0.9, 0.9, and 3.1 cm in east, north and up components, respectively.

**Figure 13:**
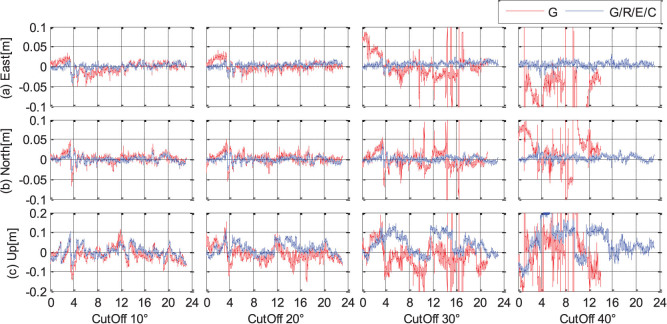
Kinematic PPP results (with backward smoothing) in single- and multi-system modes under different cut-off elevation angles (from 10° to 40°) at station CUT0 (GPS Time). The GPS-only and four-system solutions are shown by the red and blue lines, respectively.

We also analyzed the kinematic PPP performance in single- and multi-system modes under different cut-off elevation angles, ranging from 10° to 40°. The position series of the station CUT0 are shown in Figure 13 as a typical example. The GPS-only PPP results are shown by the red lines, while the four-system PPP solutions are shown by the blue lines. We can find that the accuracy and reliability of GPS-only PPP decreases dramatically as the cut-off elevation angle increases. When the cut-off elevation is increased to 30° or 40°, the GPS-only PPP cannot provide continuous precise positioning, the PPP results are very unreliable and precise position estimates are frequently not available. However, the positioning accuracy of multi-system PPP is nearly not decreased and few centimeter are still achievable in horizontal components even with 40° cut-off elevation. The multi-PPP vertical accuracy decreases gradually as the cut-off elevation angle increases, but much better than that of single-system solutions.

**Figure 14:**
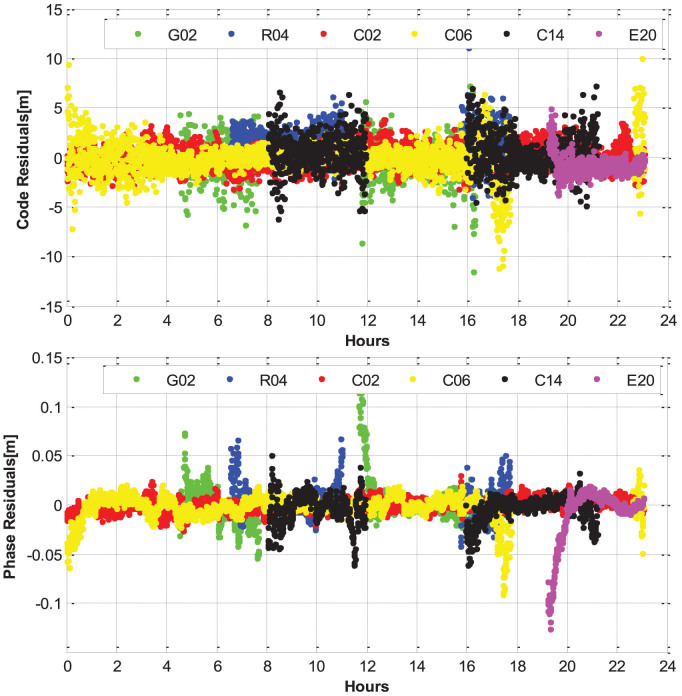
The code and phase residuals of several typical satellites from different navigation satellite systems and orbital types [G02 (GPS), R04 (GLONASS), C02 (BeiDou GEO), C06 (BeiDou IGSO), C14 (BeiDou MEO), and E20 (Galileo)], on September 3, 2013 (GPS Time).

**Figure 15:**
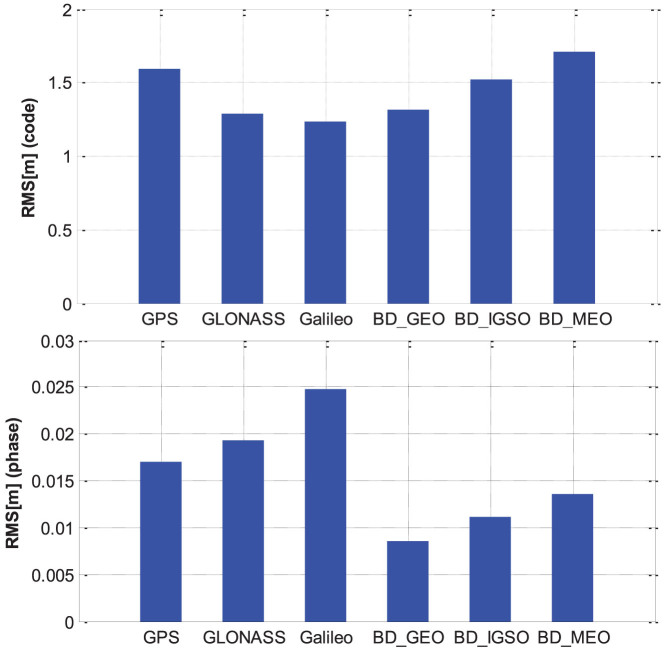
The RMS values of phase and code residuals (MGEX and BETN networks from September 1 to December 9 in 2013) for different satellite systems and orbital types (GPS, GLONASS, Galileo, BeiDou GEO, BeiDou IGSO, and BeiDou MEO).

Observation residuals, which mainly contain the observation noises, multipath, and other errors that are not fully modeled, can also be used as an important index to assess the observation quality and positioning accuracy. Figure 14 shows the phase and code residuals of several typical satellites from different satellite systems and orbital types. It can be seen that the phase residuals are generally within ±2.0 cm and the code residuals are generally within ±3.0 m, except some low-elevation periods. The large errors of the code observations will not influence the positioning results significantly as the weighting of code observations is much smaller (usually about 1:10,000) than that of phase observations. Figure 15 shows the RMS values of the phase and code residuals for different satellite systems and orbital types. The RMS value of GPS code residuals is about 1.6 m. The RMS value of GLONASS code residuals is about 1.3 m and smaller compared to GPS. This conclusion is different from the findings in some other publications, e.g., Cai and Gao (2013)^10^. These authors found that the GLONASS residuals are much larger compared to GPS. It can be explained by the fact that the GLONASS inter-frequency biases are well considered in our multi-system positioning model. In addition, the inter-frequency bias parameters can also absorb the systematic part of code errors to some extent. The RMS value of Galileo code residuals is smallest and about 1.2 m, while the RMS value of BeiDou MEO's code residuals is largest and about 1.7 m. For the different orbital types of BeiDou satellites, the RMS of GEO residuals is smallest and about 1.3 m, while the RMS of the IGSO residuals is about 1.5 m, which is between that of GEO and MEO. It is similar to the situation of MPCs and the possible reason is that the proportion of low-elevation periods for IGSO is lower than that of MEO and the GEO satellites have the lowest low-elevation proportion as their stable elevations within service regions. For the BeiDou phase observations, the RMS of the GEO residuals is also smallest and about 0.9 cm, while the RMS of MEO residuals is also largest and about 1.4 cm. We can also find that the RMS value of BeiDou MEO residuals is even slightly smaller than GPS residuals, which is about 1.7 cm. The GLONASS residuals are slightly larger than GPS ones and the corresponding RMS is about 1.9 cm. The Galileo residuals have the largest RMS of 2.5 cm, which can be caused by some modeling parameters that are not accurate enough (e.g. phase center offset and variation values).

## Discussion

In order to take full advantage of the current available GNSSs, we developed a BeiDou + Galileo + GLONASS + GPS four-system positioning model to fully exploit all currently available GNSS observations. With the multi-GNSS data from the MGEX and BETN ground tracking networks, we first fully analyzed and compared the visibility, PDOP, SNR and MPC of the different constellations with real data. Precise positioning is then performed and the results show that the addition of BeiDou, Galileo and GLONASS systems to the standard GPS-only processing significantly shorten the convergence time and improve the positioning accuracy. Meanwhile, the position series of multi-PPP are much more stable than GPS-only solutions, with much smaller and fewer fluctuations. Some spikes, appeared in single-system solutions, can be easily solved when multi-GNSS observations are used together. The accuracy and reliability of GPS-only PPP decreases dramatically when the cut-off elevation angle increases, especially in kinematic applications. However, the accuracy of multi-PPP is not obviously affected by high cut-off elevation and few centimeters are achievable even with 40° cut-off elevation. In kinematic multi-PPP, the vertical accuracy decreases gradually as the cut-off elevation angle increases, but it is much better than that of single-system solutions.

The fusion of multiple GNSS can significantly increase the number of observed satellites, optimize the spatial geometry and improve convergence, accuracy, continuity and reliability of precise positioning. Especially, the high cut-off elevation capability of multi-GNSS will significantly increase its applicability in constrained environments, such as e.g. in urban canyons, open pits or when serious low-elevation multipath or ionospheric scintillations are present. In the sequential studies, the three-frequency observation data should be fully exploited to further improve the multi-GNSS performance. In addition, the multi-GNSS not only enhances precise positioning applications, but also offers an increased number of signals for GNSS based remote sensing as troposphere/ionosphere sounding with ground based and satellite based (radio occultation) techniques with numerous applications in atmosphere science and operational weather and space weather forecast^24^,^25^.

## Methods

The GNSS observation equations for carrier phase L and pseudorange P respectively, can be expressed as following:
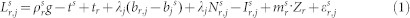

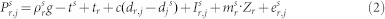

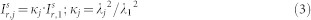
where indices *s*, *r*_, _and *j* refer to the satellite, receiver, and carrier frequency, respectively; *t*^s^ and *t_r_* are the clock biases of satellite and receiver; 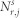 is the integer ambiguity; *b_r,j_* and 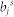 are the receiver- and satellite-dependent uncalibrated phase delay^20^ (UPD); *λ_j_* is the wavelength; *d_r,j_* and 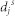 are the code biases of the receiver and the satellite; 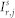 is the ionospheric delay of the signal path at frequency *j*, the ionospheric delays at different frequencies can be expressed as Eq (3); *Z_r_* is the tropospheric zenith wet delay at the station *r*, the slant tropospheric delay consists of the dry and wet components and both can be expressed by their individual zenith delay and mapping function, the tropospheric delay is usually corrected for its dry component with an a priori model, while the residual wet part of the tropospheric delay is estimated from the observations; 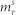 is the wet mapping function; 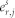 and 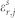 denote the sum of measurement noise and multipath error for the pseudorange and carrier phase observations. Furthermore, *ρ_g_* denotes the geometric distance between the phase centers of the satellite and receiver antennas at the signal transmitting and receiving time, respectively. This means, that the phase center offsets and variations and station displacements by tidal loading must be considered. Phase wind-up and relativistic delays must also be corrected according to the existing models^21^, although they are not included in the equations.

Usually, the ionosphere-free linear combination is used for PPP to eliminate the ionospheric delays^21^. In this contribution, we use the raw carrier phase and pseudorange observations of Eqs. (1), (2), and (3) and estimate the slant ionospheric delay as unknown parameters^22^. The linearized equations for (1) and (2) can be respectively expressed as following,
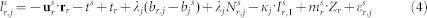

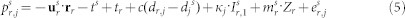
where 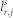 and 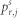 denote “observed minus computed” phase and pseudorange observables from satellite *s* to receiver *r* at the frequency *j*; 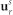 is the unit vector of the direction from receiver to satellite; **r**_*r*_ denotes the vector of the receiver position increments relative to a priori position which is used for linearization. For the multi-constellation case, the combined BeiDou + Galileo + GLONASS + GPS observation model can be formulated as,
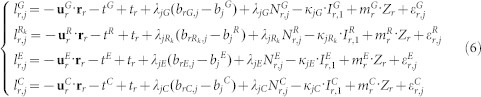

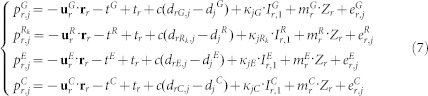
where indices *G*, *R*, *E* and *C* refer to the GPS, GLONASS, GALILEO, and BeiDou satellites, respectively; *R_k_* denotes the GLONASS satellite with frequency factor k that are used for the computation of the carrier phase frequencies of the individual GLONASS satellites; *d_rG_*, *d_rR_*, *d_rE_*, and *d_rC_* denote the code biases of the receiver *r* for *G*, *R*, *E* and *C*, respectively. Because of the different frequencies and signal structure of the individual GNSS, the code bias values *d_rG_*, *d_rR_*, *d_rE_*, and *d_rC_* are different in one multi-GNSS receiver. The differences between them are usually called inter-system biases (ISB) for code observations. Similarly, the phase delays *b_rG_*, *b_rR_*, *b_rE_* and *b_rC_* are also different and their differences are inter-system biases for phase observations; As GLONASS satellites emit the signals on individual frequencies, it will also lead to frequency-dependent biases in the receivers. For the GLONASS satellites with different frequency factors, the receiver code bias *d_rR_*, as well as phase delay *b_rR_*, are different. Their differences are usually called inter-frequency biases (IFB).

The inter-system and inter-frequency biases must be considered in a combined processing of multi-GNSS observations. The code bias parameters are setup for each system and each frequency of GLONASS. In order to eliminate the singularity between receiver clock and code bias parameters, the code bias for GPS satellites is set to zero. This means that all estimated biases of other systems are relative to the biases for the GPS satellites. These estimated biases can be interpreted as a relative calibration of “BeiDou/Galileo with respect to GPS” and “each individual frequency used by a GLONASS satellite with respect to the GPS frequency”. It is worthwhile to notice that such a receiver internal bias is only relevant for processing the code data. When analyzing the phase measurements the corresponding phase ambiguity parameters will absorb the phase delays. They become only relevant if ambiguities between different GNSS are resolved to their integer values.

For precise positioning users, precise satellite orbit, clock and DCB products (e.g. from MGEX products) need to be applied. With these products, the positioning model then can be simplified as,


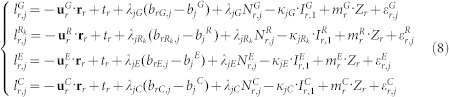

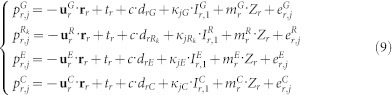
In multi-GNSS PPP, the parameters to be estimated in the combined processing contain the receiver position increments **r**_r_, receiver clock bias *t_r_*, zenith tropospheric wet delay *Z_r_*, slant ionospheric delay 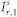, phase ambiguity 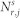, and the system/frequency dependent code biases in the receiver end, i.e. *d_rE_*, *d_rC_* and *d_rR_* relative to the GPS biases *d_rG_*. The phase delays *b_r_* and *b*^5^ will be absorbed by phase ambiguity parameters. The estimated parameters can be expressed as,
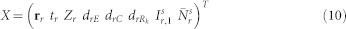

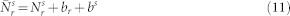
In order to strengthen the solution, a priori knowledge of the ionospheric delays including the temporal correlation, spatial characteristics and external ionospheric model is also utilized to constrain the estimated ionospheric parameters^22^,^23^. These constraints, to be imposed on observations of a single station can be summarized as,
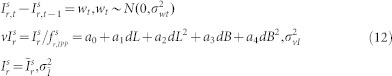
where *t* is the current epoch and *t* − 1 is the previous epoch; *w_t_* is a zero mean white noise with variance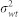; 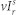 is the vertical ionospheric delay with a variance of 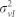; 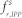
is the mapping function at the ionospheric pierce point (IPP); the coefficients *a_i_* describe the trend; *dL* and *dB* are the longitude and latitude difference between the IPP and the station location; 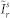 is the ionospheric delay obtained from external ionospheric model with a variance of 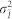.

Table 3 summarizes our multi-GNSS data processing strategy in detail. In our PPP model, all the observations from different GNSS (four systems) are processed together in one common least square estimator. The raw-observation model with ionospheric constraints of equation (12) is adopted to improve the PPP performance. The receiver positions are estimated in both static and kinematic modes. The tropospheric zenith wet delay *Z_r_* is described as a random walk process with noise of about 
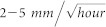
. The receiver clock is estimated epoch-wise as white noise. The carrier-phase ambiguities are estimated as constant for each arc. The ISB and IFB parameters are estimated as constant over time and GPS is selected as reference.

## Author Contributions

X.L. and X.Z. initial idea and conception; X.L. and X.R. wrote the main manuscript text; M.F., J.W. and H.S. helped with the writing. All authors reviewed the manuscript.

## Figures and Tables

**Table 1:** The RMS values of static PPP solutions with different session lengths (15 min, 30 min, 1 h, 2 h, 4 h, 6 h and 12 h) in single- and multi-system modes (MGEX and BETN networks from September 1 to December 9 in 2013)

System	Direction	0.25h	0.5 h	1.0 h	2.0 h	4.0 h	6.0 h	12.0 h
**G (cm)**	E	15.75	10.56	4.18	1.61	0.88	0.72	0.27
	N	6.29	2.37	1.19	0.48	0.30	0.16	0.14
	U	14.19	9.99	5.95	1.63	1.25	1.07	0.49
**R (cm)**	E	14.24	14.77	6.37	2.86	0.62	0.28	0.25
	N	14.19	10.98	4.10	1.43	0.30	0.22	0.12
	U	14.28	12.32	6.92	1.83	1.03	1.04	0.53
**C (cm)**	E	16.09	15.83	16.62	11.38	7.68	6.15	1.56
	N	13.13	11.04	14.43	9.41	3.17	3.11	0.44
	U	12.14	16.26	18.36	13.82	8.64	7.19	1.81
**G/R/E/C (cm)**	E	7.03	2.17	1.52	0.65	0.37	0.24	0.17
	N	2.51	1.02	1.07	0.43	0.18	0.14	0.11
	U	7.90	3.00	2.33	1.38	0.72	0.66	0.32

**Table 2:** The RMS values of kinematic PPP solutions of single- , and four-system modes in north, east and up components (MGEX and BETN networks from September 1 to December 9 in 2013)

System	E(cm)	N(cm)	U(cm)
**G**	1.4	1.2	4.4
**R**	2.9	1.8	5.9
**C**	1.3	1.3	5.4
**G/R/E/C**	0.9	0.9	3.1

**Table 3:** Data processing strategy and model for multi-GNSS PPP

Item	Strategies & Models
**Estimator**	All the Multi-GNSS observations are processed together in one common least square estimator
**Observations**	Raw carrier phase and pseudorange observations; BeiDou + Galileo + GLONASS + GPS, 74 satellites in orbits
**Signal selection**	GPS: L1/L2; GLONASS: L1/L2; BeiDou: B1/B2; Galileo: E1/E5a
**Sampling rate**	30 s
**Elevation cutoff**	7°
**Weight for observations**	Elevation dependent weight
**Satellite orbit**	Fixed
**Satellite clock**	Fixed
**Earth rotation parameter**	Fixed
**Phase-windup effect**	Corrected
**Station displacement**	Solid Earth tide, pole tide, ocean tide loading IERS Convention 2003
**Satellite antenna phase center**	Corrected using MGEX and IGS values
**Receiver antenna phase center**	Corrected
**Station coordinates**	Estimated in both static and kinematic modes
**Phase ambiguities**	Estimated as constant for each arc
**Tropospheric delay**	Initial model + random-walk process
**Ionospheric delay**	Estimated as parameters with constraints of equation (12)
**Receiver clock**	Estimated, white noise
**ISB and IFB**	Estimated as constant, GPS as reference
